# Achievements and challenges of copper‐based single‐atom catalysts for the reduction of carbon dioxide to C2+ products

**DOI:** 10.1002/EXP.20230011

**Published:** 2023-07-26

**Authors:** Tianmi Tang, Zhenlu Wang, Jingqi Guan

**Affiliations:** ^1^ Institute of Physical Chemistry, College of Chemistry Jilin University Changchun P. R. China

**Keywords:** active site, carbon dioxide reduction, Faraday efficiency, single‐atom copper catalyst, stability

## Abstract

Copper is the only metal that can convert CO_2_ into C2 and C2+ in electrocatalytic carbon dioxide reduction (CO_2_RR). However, the Faraday efficiency of CO_2_ conversion to C2 and C2+ products at high current densities is still low, which cannot meet the actual industrial demand. Here, the design methods of single‐atom copper catalysts (including regulating the coordination environment of single‐atom copper, modifying the carbon base surface and constructing diatomic Cu catalysts) are reviewed, and the current limitations and future research directions of copper‐based single‐atom catalysts are proposed, providing directions for the industrial conversion of CO_2_ into C2 and C2+ products.

## Graphical abstract

The design strategies of single‐atom copper catalysts are briefly summarized and the key factor for their application in industrially electrochemical conversion of CO_2_ into C2 and C2+ products are pointed out.

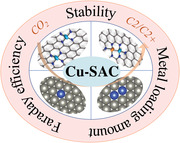

## 1 INTRODUCTION

**FIGURE 1:**
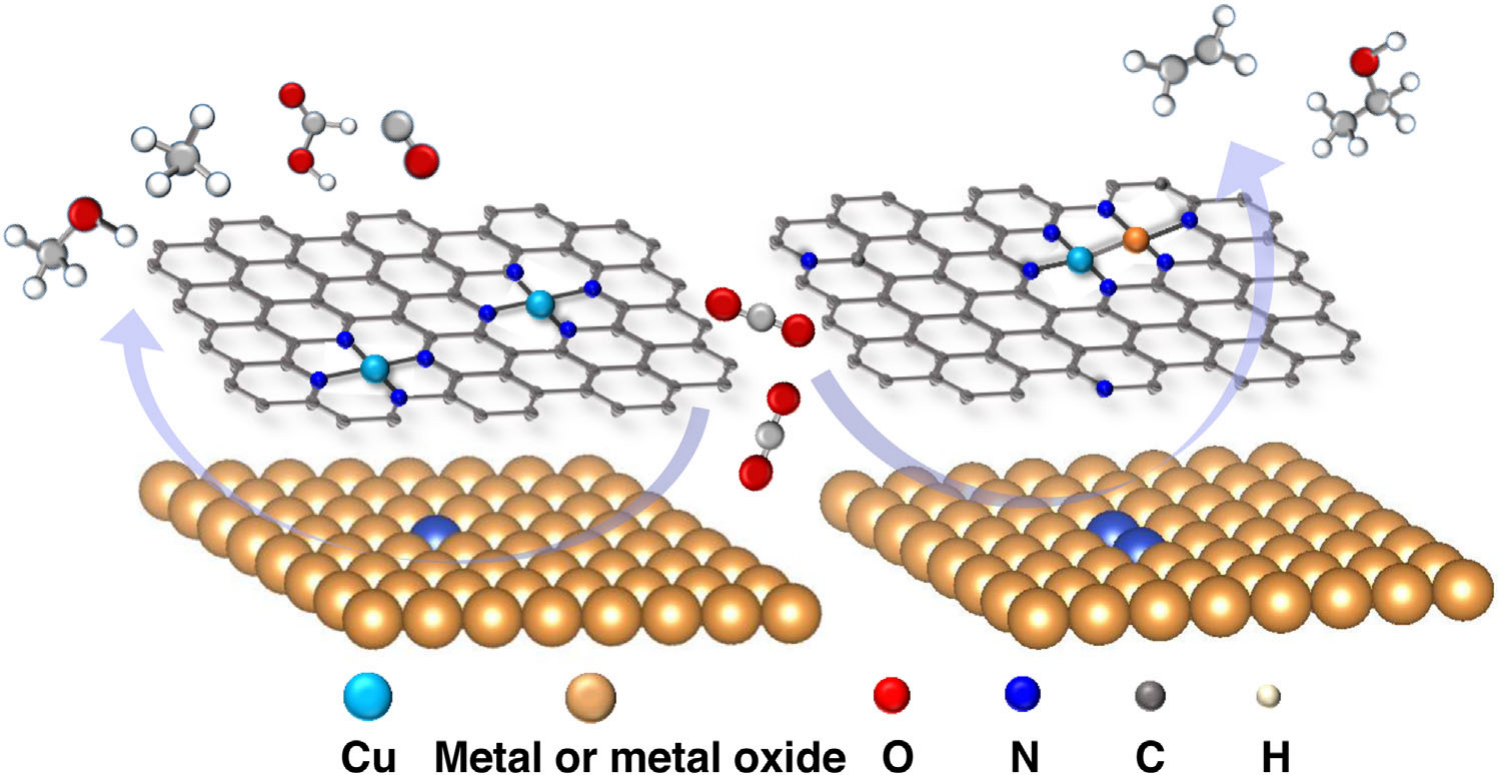
Structure diagram of copper‐based single‐/dual‐atom catalysts.

The development of highly efficient electrocatalysts for CO_2_RR is a key for mitigating global warming and providing inexhaustible energy. At present, most CO_2_RR catalysts tend to convert CO_2_ into C1 products, and there is still a large development space for generating C2+ compounds with high‐added value. In addition, for electrocatalytic CO_2_RR, the current density is not enough to meet the industrial application. Copper is the only metal that can convert CO_2_ to C2 and C2+ in electrocatalytic CO_2_RR as reported so far.^[^ ^1^ ^]^ For single‐atom catalyst (SAC), the use of metal atoms can be maximized and the density of the active site can be effectively increased. The unique coordination environment and electronic structure of metal atoms are conducive to improving the reactivity of the active site. In the synthesis of copper‐based SACs, to prevent the migration and aggregation of atomically dispersed metal atoms with high surface energy, the general synthesis methods can be divided into two categories; one is uniform dispersion of metal species into the whole substrate, such as the anchoring of Cu atoms on graphene, carbon nanotubes, nitrogen carbide, and metal‐organic frameworks and their derived carbon materials. Different structures of Cu─N─C and Cu─M─N─C can be obtained (Figure 1 and Table 1).^[^ ^2^ ^]^ The other is atomic surface engineering, such as depositing Cu atoms on the surface of metal, metal oxide, and metal sulfide by atomic layer deposition.^[^ ^3^ ^]^

**TABLE 1:** Summary of CO_2_RR performance on Cu‐based single‐atom electrocatalysts.

Catalyst	Active site	Electrolyte	Product	FE (%)	TOF (s^−1^)	Ref.
PorCu	Cu–N_4_	0.5 m KHCO_3_	CH_4_, C_2_H_4_	54	–	^[^ ^2b^ ^]^
CuPc	Cu–N_4_	0.5 m KHCO_3_	C_2_H_4_	25	–	^[^ ^2c^ ^]^
Cu–N–C‐900	Cu–N_4_	0.1 m KHCO_3_	CH_4_	38.6	–	^[^ ^2d^ ^]^
Cu–N–C‐800	Cu–N_2_	0.1 m KHCO_3_	C_2_H_4_	24.8	–	^[^ ^2d^ ^]^
Cu‐N‐C	Cu–N_4_	0.1 m CsHCO_3_	C_2_H_5_OH	55	–	^[^ ^2e^ ^]^
Cu‐SA/NPC	Cu‐pyrrolic‐N_4_	0.1 m KHCO_3_	CH_3_COCH_3_	36.7	–	^[^ ^4^ ^]^
Cu SACs	Cu–N_4_	0.1 m KHCO_3_	CO	98	–	^[^ ^5^ ^]^
Cu‐CDs	CuN_2_O_2_	0.5 m KHCO_3_	CH_4_	78	0.66	^[^ ^6^ ^]^
Fe/Cu–N–C DAC	N_4_Fe–CuN_3_	0.1 m KHCO_3_	CO	99.2	1.4	^[^ ^2i^ ^]^
Ni/Cu–N–C	NiN_4_–CuN_4_	0.5 m KHCO_3_	CO	99.2	1.9	^[^ ^7^ ^]^
BIF‐102NSs	Cu_2_	0.5 m KHCO_3_	C_2_H_4_	11.3	–	^[^ ^8^ ^]^
Cu/C‐0.4	Cu_3_	0.1 m KHCO_3_	C_2_H_5_OH	91	0.037	^[^ ^2f^ ^]^
Cu/p‐Al_2_O_3_ SAC	Cu SA	1 m KOH	CH_4_	62	–	^[^ ^3c^ ^]^
Cu/Ag_2_S/Ag	Cu/Ag(S)	0.1 m KHCO_3_	C2+	∼17	–	^[^ ^3a^ ^]^

To improve the efficiency of single‐atom copper catalysts in CO_2_RR, diatomic copper catalysts can be constructed by adjusting the coordination environment of copper atoms. The electron structure and coordination environment of metal atoms and the electron interaction between the metal–ligand moiety and the supporting carrier can be adjusted to affect the adsorption behavior of key reaction intermediates. For example, Cu─C can be constructed in the graphdiyne (GDY) base plane, which provides a channel for charge transfer and promotes the charge transfer. The formed hybrid orbital between Cu and C causes the change of electronic structure of isolated copper atoms. The ─C≡C─C≡ structure in GDY can effectively stabilize isolated single Cu atoms and prevent them from grouping. The Cu─C bond favors the formation of *OCHO intermediate and improves the selectivity of CH_4_.^[^ ^9^ ^]^ The delocalized π bond on the base plane interacts with the d orbital of Cu to affect the electronic structure of the active site of Cu─N*_x_*. In addition, the carbon carrier can be modified by adjusting the grain size of the carbon plane, introducing heteroatoms on the carbon plane, and constructing defects.^[^ ^6^ ^]^ Metal and metal oxides are also good carriers of atomic Cu catalysts. The interaction between them is conducive to the reduction of metal types, so as to change the adsorption performance of the two reactants (CO_2_ and H_2_) in the reaction, such as Cu loading onto ZrO_2_, CeO_2_, and other oxides.^[^ ^10^ ^]^ Heteronuclear or heteronuclear diatomic catalysts formed by Cu atoms can improve the faraday efficiency (FE) of CO_2_ conversion to C2 products. Due to the interaction between the two metals resulting in changes in electronic and geometric structure, bimetals can initiate C─C coupling and stabilize oxygen‐containing intermediates through linking effects.^[^ ^11^ ^]^

**FIGURE 2:**
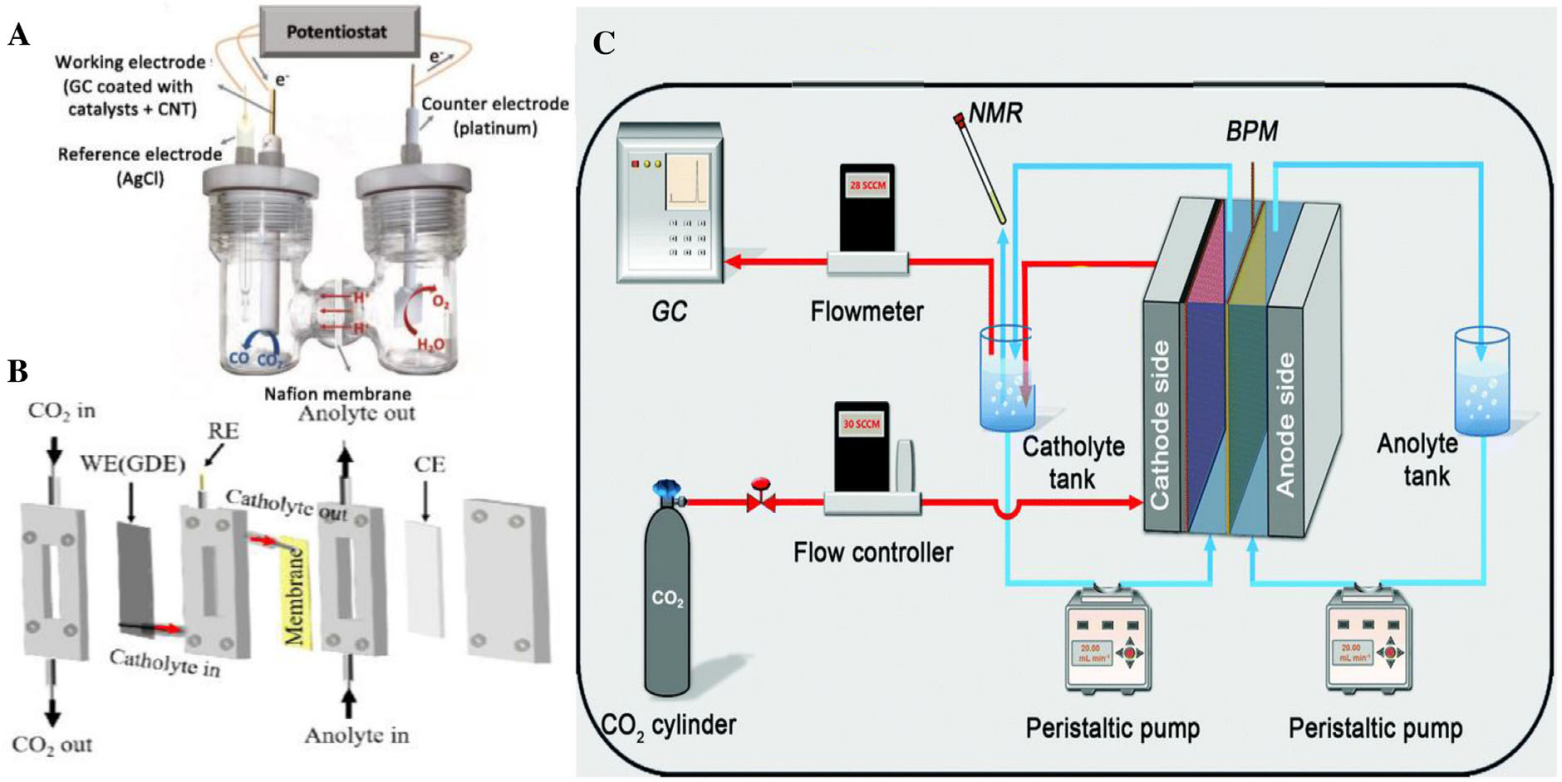
Types of CO_2_ electrolytic cells. (A) Schematic for H‐cell setup. (B) Schematic for flow cell setup. (C) Flow‐cell measurement systems. (A) Reproduced with permission.^[^ ^13^ ^]^ Copyright 2020, Elsevier B.V. (B) Reproduced with permission.^[^ ^14^ ^]^ Copyright 2023, American Chemical Society. (C) Reproduced with permission.^[^ ^12^ ^]^ Copyright 2021, The Royal Society of Chemistry.

Currently, CO_2_ electrolytic cells are mainly divided into H‐type electrolytic cells and flow‐type electrolytic cells (Figure 2A, B). Compared with H‐type electrolytic cells, flow‐type electrolytic cells can improve the performance of CO_2_RR and realize the transition from laboratory to industrialization. The designed electrolytic cells should be able to transfer the required amount of CO_2_ to the cathode catalyst. The following problems should be considered in the design of the flow cell. The CO_2_ flow rate of the flow cell can be measured to avoid bubbles gathering and being carried away by the cathode liquid under the high rate of CO_2_ electrolysis, and to avoid the cross between the cathode and the anode of the liquid products and the evaporation of the liquid products. The mass flowmeter can be added to the flow pool to detect the CO_2_ flow rate, and the gas product can be fully collected by adjusting the flow rate (Figure 2C). In addition, bipolar membranes are used to inhibit electromigration and electro‐osmotic resistance to avoid crossover problems. In practical applications, energy efficiency, collection of liquid products, and bipolar membrane (BPM) should also be considered in order to reduce CO_2_ more economically.^[^ ^12^ ^]^

To reach the entry level of the market, CO_2_ electrolytic cells must meet the requirements of high current density and selectivity as well as various indicators, such as current efficiency, battery voltage, product output concentration, and service life. Therefore, the designed catalyst should have high activity and selectivity, so as to ensure that the electrolytic cell can work for a long time. Cu‐based single‐atom electrocatalysts are expected to be applied to CO_2_ electrolytic cells. By adjusting the structure, catalysts with high current density, high selectivity, and long tolerance can be synthesized by selecting suitable substrate and coordination environment.^[^ ^15^ ^]^ For example, Wang et al. anchored single‐atom Cu to commercial Al_2_O_3_‐Lewis acid with a 62% selectivity of CH_4_ and a current density of 153.0 mA cm^−2^ at −1.2 V.^[^ ^3c^ ^]^ In this perspective, the limitations and development directions of atomic copper catalysts are mainly discussed from three aspects: stability, metal loading amount, and FE.

## 2 LIMITATIONS AND DEVELOPMENT DIRECTIONS OF ATOMIC COPPER CATALYSTS

### 2.1 Stability

**FIGURE 3:**
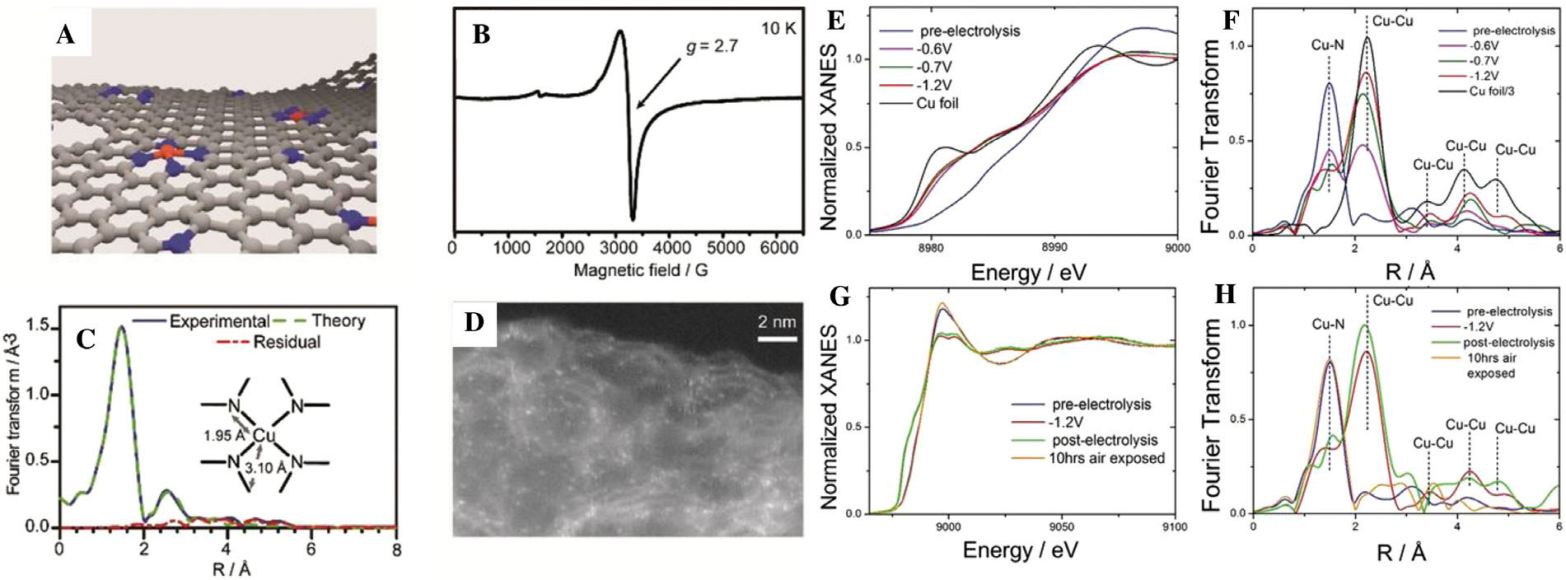
Structural characterization of Cu─N─C. (A) Structure of CuN_4_. (B) Electron paramagnetic resonance spectrum of Cu_0.5_NC. (C) Cu K‐edge extended X‐ray absorption fine structure (EXAFS) analysis in the Fourier‐transformed space. (D) High‐angle angular dark field‐scanning transmission electron microscopy image. (E) K‐edge X‐ray absorption near edge structure (XANES) spectra of Cu_0.5_NC. (F) Fourier transform of the experimental EXAFS spectra. (G) Comparison between the K‐edge XANES experimental spectrum. (H) Fourier transform of the experimental EXAFS spectra. Reproduced with permission.^[^ ^2e^ ^]^ Copyright 2019, Wiley‐VCH Verlag GmbH & Co. KGaA, Weinhem.

The stability of catalyst includes mechanical stability and reaction stability. Catalysts can be loaded or in situ synthesized onto carbon sheets, carbon paper, nickel foam, copper foam, and other support materials to reduce their mechanical detachment. Due to high utilization rate of atoms, the increase of surface free energy and highly unsaturated coordination environment, the mobility of single atoms on the carrier surface increases. In the catalytic process, isolated metal atoms will gradually form clusters and nanoparticles, which will lead to the reduction of active sites, and in serious cases, catalyst deactivation.^[^ ^16^ ^]^ Xu et al. synthesized a carbon‐supported copper catalyst. The Faraday efficiency (FE) of ethanol in CO_2_RR is highly sensitive to the structure, which is significantly reduced when monatomic Cu is converted into CuO and Cu clusters.^[^ ^2f^ ^]^ The current measures to improve the stability of single‐atom catalysts include reducing the mobility of single metal atoms by using space restriction, modifying adjacent sites to form strong bonding between metal and other atoms, and inhibiting metal migration by using the interaction between metal and support. The activity and stability of the catalyst are improved by anchoring a single atom at high temperature and regulating the electronic properties of a single atom with the help of charge transfer by the interaction of electron metal‐carrier.^[^ ^4^, ^17^ ^]^ Fontecave et al. synthesized a Cu─N‐doped carbon material (Cu─N─C). The Cu─N─C catalyzed the conversion of CO_2_ to ethanol with a FE of up to 55%, and ethanol was the only liquid phase product, in which CO was the key intermediate. The results of X‐ray absorption spectroscopy (XAS) showed that the CuN_4_ active site underwent instantaneous transformation during the electrochemical reaction. The active site underwent recombination and transformation into copper nanoparticles, which was reversible and then transformed back to CuN_4_ (Figure 3).^[^ ^2e^ ^]^

### 2.2 Metal loading amount

**FIGURE 4:**
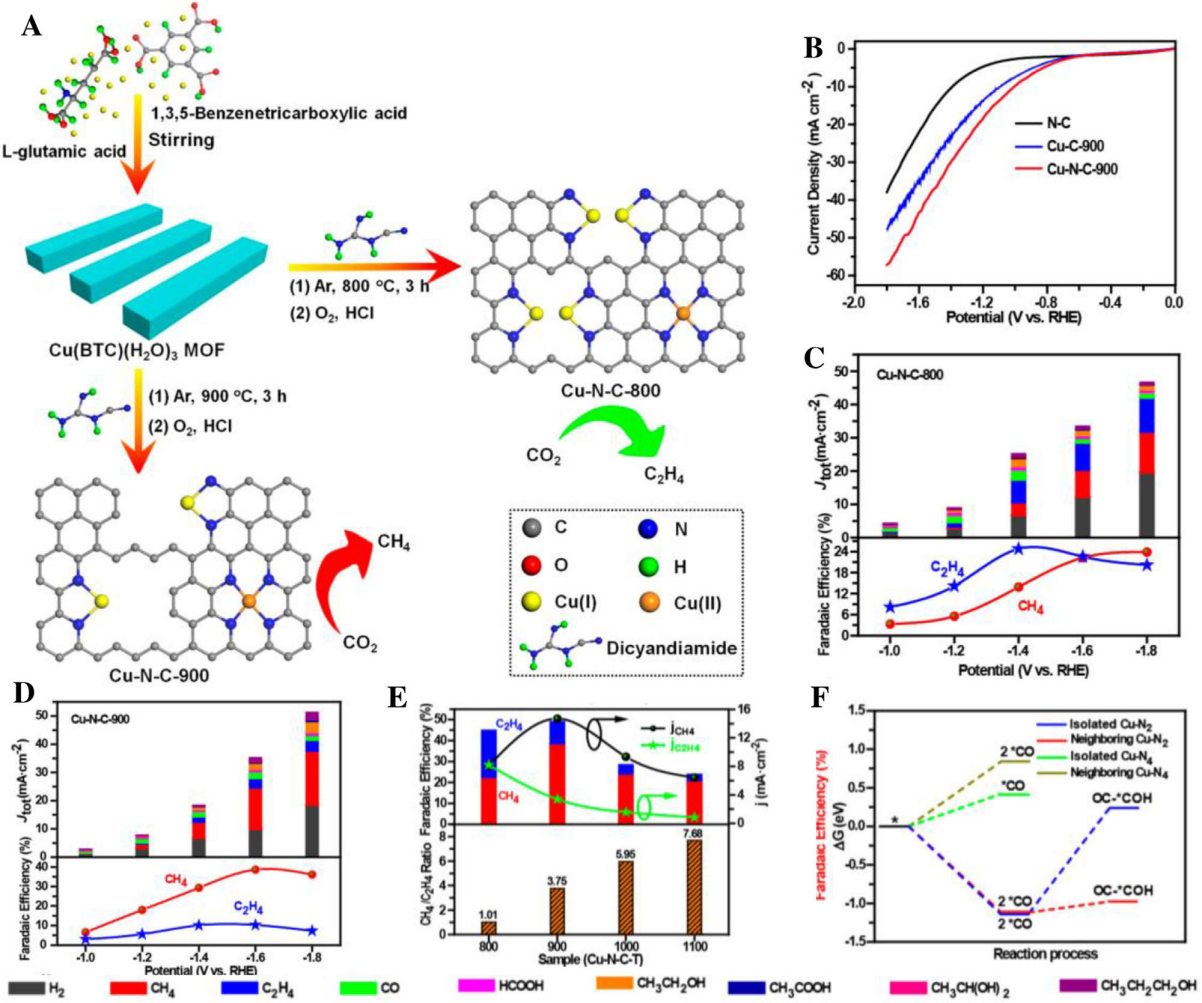
Synthesis, performance comparison diagram and theoretical analysis of Cu─N─C‐T catalysts. (A) Synthetic procedure of Cu─N─C‐T catalysts. (B) Linear sweep voltammetry curves. (C,D) CO_2_ electroreduction performance of Cu─N─C‐800 and Cu─N─C‐900. (E) Faradaic efficiencies, partial current densities of CH_4_ and C_2_H_4_, and the ratios of CH_4_/C_2_H_4_ at −1.6 V. (F) Calculated Δ*G* for CO_2_ electroreduction on different Cu–N*_x_*. Reproduced with permission.^[^ ^2d^ ^]^ Copyright 2020, American Chemical Society.

Typical monatomic Cu catalysts have been prepared with low metal loading content to overcome high surface free energy to obtain highly dispersed metal atoms in space isolation state. To increase the effective active site density, it is necessary to increase the metal loading. Currently, the common way is to stabilize single metal atoms by the covalent coordination or ionic interaction between the metal and adjacent heteroatoms, or to load metal atoms into porous materials, which can increase the dispersion density of the metal. Huang et al. synthesized Cu SAC with 12 wt% Cu loaded by using C_3_N_4_ with rich nitrogen atoms.^[^ ^18^ ^]^ Mono‐/dual‐atomic catalysts have been synthesized with different adjacent active centers. Co‐catalysis of two metals or metal dimers facilitates C─C coupling and conversion of CO_2_ to C2. Zheng et al. reported that the selectivity of CO_2_RR can be affected by the concentration of Cu doping. The combination of experiment and theory testified that the proximity between Cu atoms can enhance the C─C coupling and reduce CO_2_ to ethylene when the load density of Cu was 4.9%_mol_. When the load density of copper was 2.4%_mol_, CO_2_ was mainly reduced to methane (Figure 4).^[^ ^2d^ ^]^

In addition, with increasing the metal loading capacity, it is necessary to ensure that the metal exists in the form of single‐/double‐atom structure. For example, a single‐atom Cu─Zr catalyst synthesized by Liu et al. contained copper clusters or small nanoparticles when the Cu loading content is greater than 8 wt%.^[^ ^19^ ^]^ The ultra‐fast synthesis method can reduce the aggregation of metal atoms, and the synthesis time usually takes only a few nanoseconds to a few minutes. Currently, the laser assisted synthesis, microwave assisted synthesis, Joule heating, and plasma synthesis are widely used. These technologies are characterized by rapid heating and cooling, and can be used to accurately synthesize catalysts with different structures.^[^ ^20^ ^]^

### 2.3 Faraday efficiency

**FIGURE 5:**
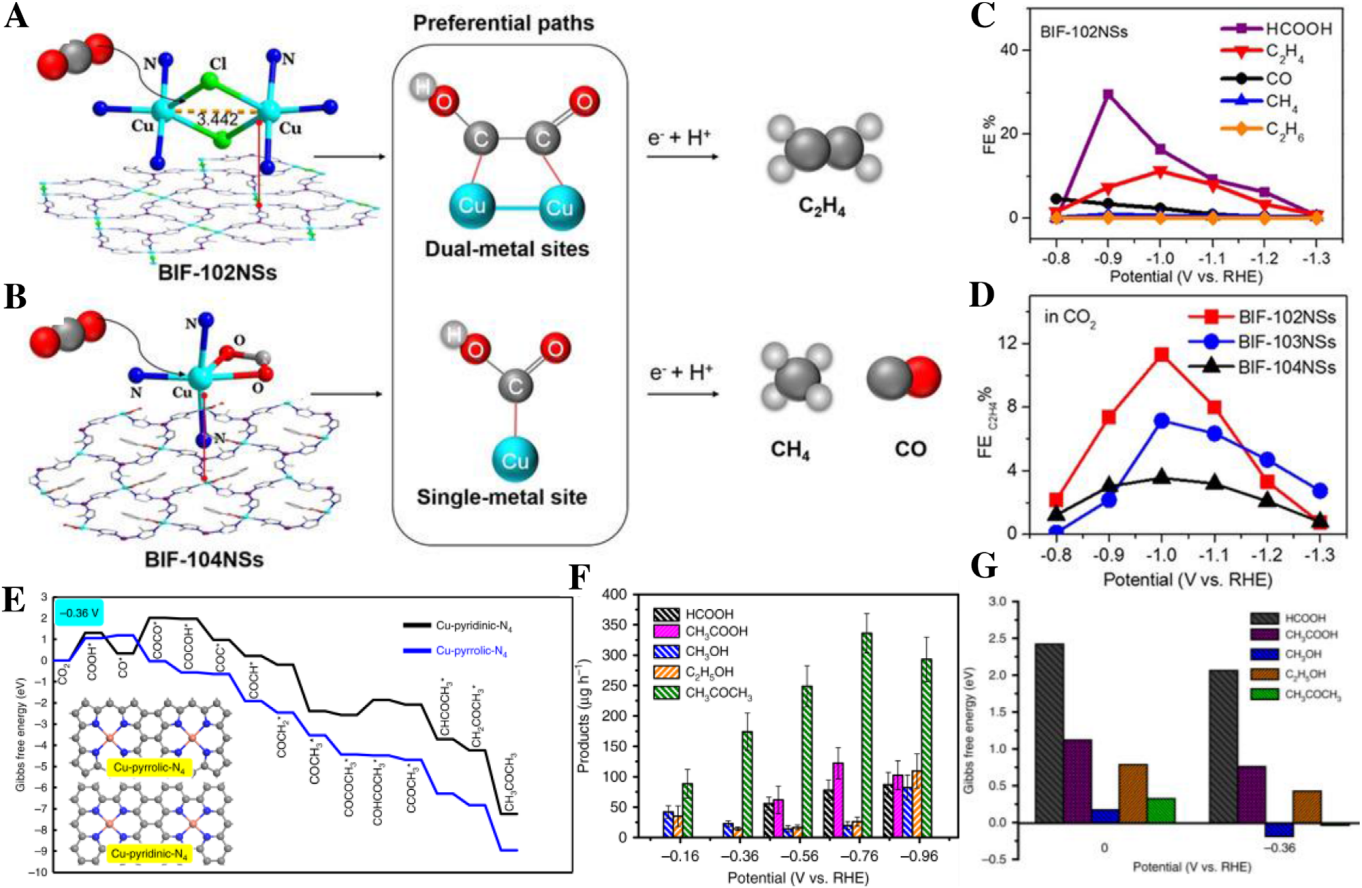
Comparison of different reaction paths and FE. Coordination environment and the preferential reaction pathways of BIF‐102NSs (A) and BIF‐104NSs (B). (C) The FE. (D) The $FE_{C_{2}H_{4}}$ . (E) Density functional theory (DFT) calculations of Cu‐SA/NPC. (F) Production rate. (G) Relative selectivity evaluation on the Cu‐pyrrolic‐N_4_ site. (A–D) Reproduced with permission.^[^ ^8^ ^]^ Copyright 2021, Wiley‐VCH Verlag GmbH & Co. KGaA. (E–G) Reproduced with permission.^[^ ^4^ ^]^ Copyright 2020, Nature Portfolio.

It is generally believed that the binding strength of potential CO intermediate and active sites determines the type of products. For example, Cr, Fe, and Co metal sites are too strongly bound to CO, and hydrogen evolution reaction (HER) is dominant, while Ni and Zn are weakly bound to CO, and CO_2_ tends to be reduced to CO; and Sn and Bi are weakly bound to CO, and CO_2_ is easily reduced to formate. Cu‐based catalysts have the best CO affinity, on which C2 and C2+ products can be generated through C─C coupling reaction.^[^ ^21^ ^]^ Zhang et al. constructed catalysts with single copper sites and double copper sites. On the double copper sites, CO_2_ was mainly reduced to C_2_H_4_; while on the single copper sites, it was mainly reduced to CH_4_. Possibly due to the synergic effect of the two sites, charge was enriched around the copper center, which was conducive to C─C coupling and changing the reaction path of CO_2_RR (Figure 5A–D).^[^ ^8^ ^]^ Chen et al. synthesized a monatomic copper catalyst coated with nitrogen‐doped porous carbon (Cu‐SA/NPC), which was mainly reduced to acetone in CO_2_RR with a FE of 36.7%. Theoretical calculations showed that Cu─N_4_ (pyrrolin) was the active site, on which the CO_2_ activation energy and the energy required for C─C coupling were reduced (Figure 5E–G).^[^ ^4^ ^]^

**FIGURE 6:**
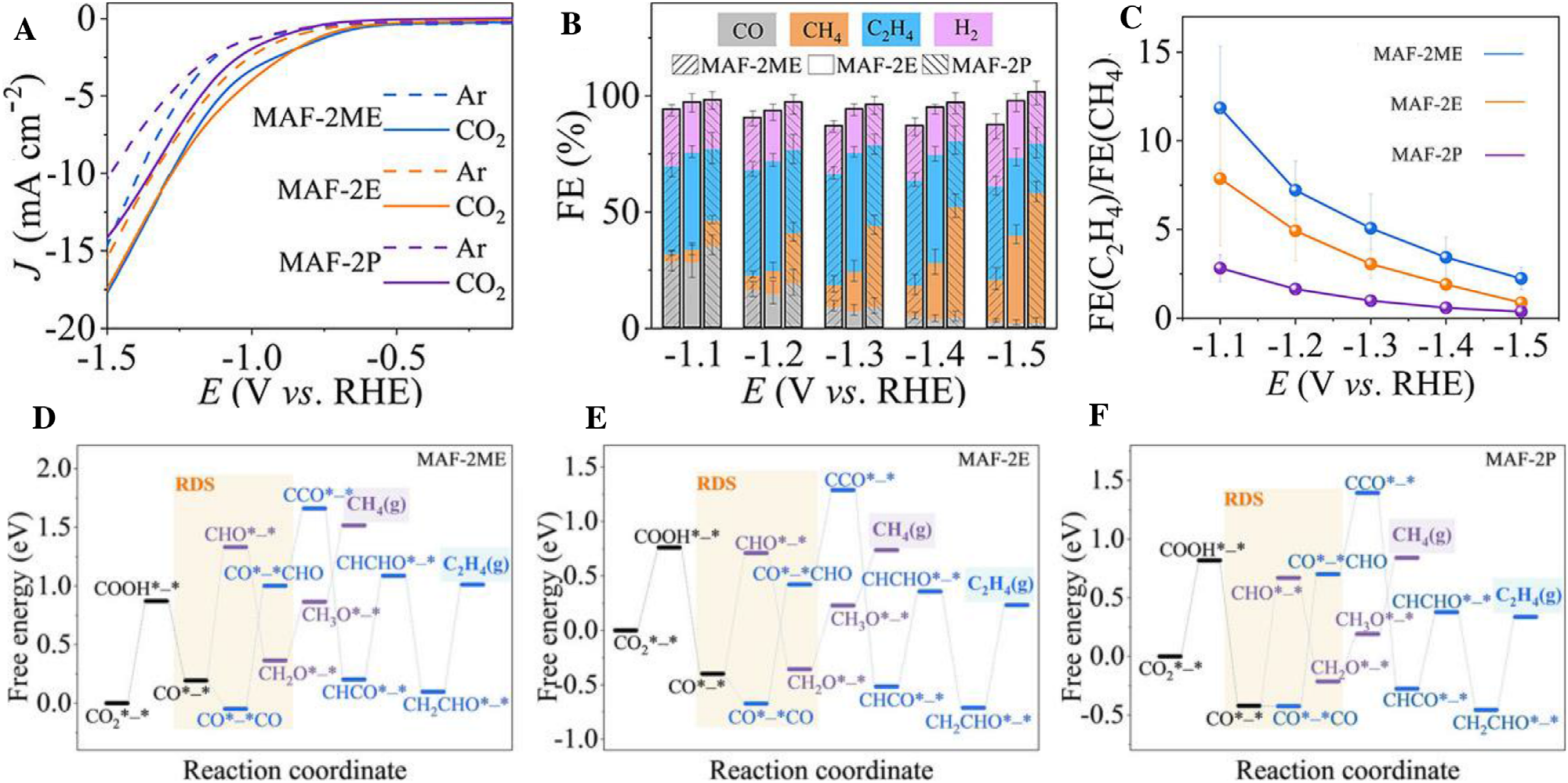
Performance comparison and theoretical calculations. (A) LSV curves. (B) Potential‐dependent FE of gas products. (C) Potential‐dependent C_2_H_4_/CH_4_ selectivity. PDFT‐derived free energy diagrams of CO_2_RR for (D) MAF‐2ME, (E) MAF‐2E, and (F) MAF‐2P. Reproduced with permission.^[^ ^24^ ^]^ Copyright 2022, Wiley‐VCH.

The reduction of CO_2_ to C2 products by C─C coupling occurs at diatomic Cu sites. The ratio of C1/C2 products can be affected by adjusting the charge density of diatomic Cu, the distance between adjacent Cu sites, and the bonding ability with the surrounding ligand.^[^ ^22^ ^]^ Metal azolate framework‐2 (MAF‐2) has good flexibility and can influence the FE in catalytic CO_2_ by adjusting non‐coordination side groups reasonably.^[^ ^23^ ^]^ Zhang et al. reported a MAF‐2 analogue with binuclear copper sites with FE of 51%, 56%, and 77% for reducing CO_2_ to C_2_H_4_, CH_4_, and hydrocarbons, respectively. The C_2_H_4_/CH_4_ ratio can be changed by adjusting the size of the non‐ligand side group. Theoretical calculation results showed that the Cu(I) coordination structure changed from triangle to tetrahedron after adsorption of intermediates. The rate determination steps of CO_2_ reduction to CH_4_ and C_2_H_4_ are CO*─* → CHO*─* and CO*─*CO─*CHO, respectively. The synergistic action of two adjacent Cu(I) promoted the formation of C_2_H_4_ through C─C coupling (Figure 6).^[^ ^24^ ^]^ However, single‐atom Cu catalysts usually have low FE in catalyzing CO_2_ to C2 and C2+ products, especially at high industrial current densities. The FE of the product can be improved through tandem action to increase the density of the active site by increasing the metal load. The unique structure of the metal dimer catalyzes the conversion of CO_2_ to C2 by triggering C─C coupling and stabilizing oxygen‐containing intermediates through the linking effect. In addition, the overpotential of C─C coupling can also be reduced on three or more atomic clusters to alleviate the highly reducing electrolytic environment and improve the FE of the product. However, diatomic and polyatomic catalysts are still in their infancy, and the synthesis strategies, the structures and catalytic properties need to be developed to obtain products with high FE at high current densities.

## 3 PERSPECTIVES

We have reviewed the recent development of Cu‐based single‐atom electrocatalysts for the generation of high value‐added C2 and C2+ products in the CO_2_RR. Catalysts with abundant active centers can be obtained by modulating the electronic structure of Cu atoms and their surroundings, so as to stabilize C2 and C2+ intermediates and promote the generation of high value‐added products. In this process, Cu atoms can be used to effectively inhibit the competitive HER. The current challenges in Cu‐based single‐atom catalysts are mainly stability, metal loading, and FE. The longest lifetime of CO_2_ to C2 reported so far can reach 200 h, which is not enough for industrial production. The high mobility of Cu atoms would cause the aggregation, recombination or shedding of the active site, and the dynamic changes of the surface will cause the decrease of activity and selectivity. The structure or form of the catalyst can be maintained by heteroatom doping or the strong interaction between the defect site carrier and metal atoms to prolong the service life. In addition, the stability of the reaction can be improved by optimizing the reactor. Due to the high surface free energy of metal atoms in the space isolation state, the aggregation of metal atoms should be overcome by increasing the metal load to increase active sites. Currently, the common strategy is to stabilize single metal atoms by forming covalent coordination between metal and carrier heteroatoms, but the occurrence of metal clusters is still unavoidable. To solve this problem, a super‐fast synthesis method has been developed. Reducing the heating time to a few minutes or even a few nanoseconds could reduce the agglomeration of metal atoms, providing directions for precise synthesis of advanced catalysts with different structures. In addition, the confinement strategies are currently a common method for controlled synthesis of atomic catalysts, in which metal precursors are encapsulated into porous materials, such as zeolite, metal–organic frameworks, and covalent organic frameworks materials, and then pyrolyzed to obtain atomically dispersed catalysts. By directional design of metal precursors, single‐atom, diatomic, and polyatomic catalysts with different coordination structures can be obtained.

The conversion of CO_2_ into C2 products is a complex multi‐step process, and it is difficult to simultaneously improve the activity and selectivity. For example, the conversion of CO_2_ into C_2_H_5_OH and C_2_H_4_ in CO_2_RR is a competitive reaction with slow kinetics. The selective conversion to C_2_H_5_OH requires that the active site be able to perform C─C coupling while inhibiting the conversion to C_2_H_4_. It is theoretically easier to convert CO_2_ to alcohol than to hydrocarbon because the hydrogenation of O is easier than the hydrogenation of C. In addition, the generation of most C2 products requires precise design of the active site and harsh reaction conditions. At present, single‐atom Cu catalysts have good FE in catalyzing CO_2_RR at low current densities. The reactivity of the active site can be improved by modulating the electron structure of metal atoms. In situ characterization can be developed to obtain the dynamic process of intermediates and accurate active sites. Although X‐ray absorption spectra can be used to obtain the structural information of catalysts, it is necessary to focus on the development of time‐resolved and spatially resolved spectra to accurately obtain the dynamic information of key intermediates and active sites in the future. The combination of in situ characterization and theoretical calculation can help us to further study the reaction mechanism and design efficient catalysts. Although the practical application of single‐atom Cu catalysts still faces great challenges, through in‐depth research and the development of advanced instruments, their industrial utilization in CO_2_RR can be hopefully realized in the future.

## CONFLICT OF INTEREST STATEMENT

The authors declare no conflict of interest.
